# Whole-Cell Bioreporter-Based Assay for Detecting Fungal-Derived β-Lactamase Inhibitors

**DOI:** 10.3390/bios15090594

**Published:** 2025-09-09

**Authors:** Raz Benou, Robert S. Marks, Alex Sivan, Esti Kramarsky-Winter, Karina Golberg, Ariel Kushmaro

**Affiliations:** 1 Avram and Stella Goldstein-Goren, Department of Biotechnology Engineering, Faculty of Engineering Sciences, Ben Gurion University of the Negev, Beer-Sheva 84105, Israel; razhil@post.bgu.ac.il (R.B.); rsmarks@bgu.ac.il (R.S.M.); esti.winter@gmail.com (E.K.-W.); karingo@post.bgu.ac.il (K.G.); 2 The Ilse Katz Centre for Nanoscale Science and Technology, Ben-Gurion University of the Negev, Beer-Sheva 84105, Israel; 3 Department of Life Sciences, Achva Academic College, Yenon 79804, Israel; 4 School of Sustainability and Climate Change, Ben-Gurion University of the Negev, Beer-Sheva 84105, Israel

**Keywords:** antibiotics, β-lactam, β-lactamase, β-lactamase inhibitors, novel bioassay bioreporter, bioluminescence

## Abstract

β-lactams are an important family of antibiotics that are prone to undergo resistance inhibition though the production of β-lactamases by some microorganisms. To combat this resistance and preserve the efficacy of β-lactam antibiotics, we developed a strategy for the discovery of such β-lactamase inhibitors. When combined with β-lactams, these inhibitors allow the antibiotics to be effective and prevent resistance. To date, the development of such combinatory drugs is limited due to the complexity of screening for new β-lactamase inhibitors. Therefore, to facilitate this development, it was essential to find sensitive assays to effectively screen for lactamase inhibitory compounds. To this end, a novel bioassay utilizing bioluminescent indicator bacteria as bioreporters was developed. The assay was first optimized using commercial antibiotics together with known β-lactamase inhibitors. Using this bioassay, we then screened for novel natural β-lactamase inhibitors derived from coral-associated fungi. We showed that the fungus *Penicillium spinulosum*, originating from the coral *Pocillopora* sp. from the Gulf of Aqaba Eilat, produced compounds with anti-β-lactamase activity. We further demonstrated that the bioreporter bacteria used here responded to the combined antibiotics and β-lactamase inhibitors in a concentration-dependent manner, indicating their usefulness for β-lactamase-inhibiting compound discovery. Future structural identification will promote the validation of this assay’s usefulness.

## 1. Introduction

The rapid spread of extensive drug-resistant (XDR) and multidrug-resistant (MDR) Gram-negative bacteria poses a significant threat to global public health. Some antibiotics, such as β-lactam antibiotics (BLAs), exert their action through cell wall synthesis disruption by inhibiting penicillin-binding proteins (PBPs) [1]. These are the most widely used group of antibiotics [2]. However, bacteria can overcome BLAs through the production of β-lactamases, enzymes that degrade the antibiotics, thus providing these bacteria with antibiotic resistance. In general, β-lactamases can be classified into four main groups based on their central catalytic domain and substrate preference, termed A, B, C, and D [3].

These extended-spectrum β-lactamases (ESBLs) pose a serious public health concern, because their rapid global spread may result in the failure of antibiotic therapy, leading to life-threatening infections [4]. The ESBL resistance is mainly sub-clustered into three enzymatic systems, TEM, SHV, and CTX-M, encoded by inter alia *bla*_TEM_, *bla*_SHV_, and *bla*_CTX-_ gene families [5]. These ESBLs may be inactivated by β-lactamase inhibitors (BLIs).

Indeed, several well-established commercial β-lactamase inhibitors (BLIs) have been developed in order to combat microorganisms that express β-lactamases [6]. These include classic inhibitors such as clavulanic acid, sulbactam, and tazobactam; two of which were developed as synthetic compounds from 6-β-aminopenicillanic acid. When used individually, these inhibitors were found to exhibit only limited antimicrobial activity; however, when used in combination with specific β-lactam antibiotics (BLA-BLIs), they effectively reduce the minimum inhibitory concentrations (MICs) of the antibiotics needed against various pathogens [3]. The mechanism of action for these combinations involves the recognition of the inhibitors as substrates by the β-lactamase enzymes. The active site of the enzyme attacks the β-lactam ring of the inhibitor, leading to the opening of the five-membered ring. The outcome of this process depends on the energy barrier and the stability, resulting in three possible scenarios: irreversible inactivation of the enzyme, regeneration of β-lactamase activity, or temporary inactivation [7].

To overcome antibiotic resistance of β-lactam antibiotics, there is a growing market demand to develop additional β-lactam/β-lactamase inhibitor combinations. Although numerous natural products have been screened in recent years, clavulanate has been described as the most efficient natural BLI compound to date.

This has led to the search for novel active compounds from natural sources. To this end, we returned to the marine environment, and in particular coral reefs, as they are important sources of novel biologically active chemicals [8]. Since microorganisms are abundant in the marine environment, both in the water column and sediment, as well as on live substrates [9], many organisms living in these environments have developed ways of gardening antibiotic-producing microorganisms to minimize the effects of possible pathogens. Indeed, many of these microorganisms have been reported to produce metabolites that are being enlisted to develop novel medications in this post-antibiotic era [8,10]. The search for natural products as novel BLIs is an ongoing process. The identification of approximately 35,000 marine natural products, with an annual addition of over 1500 new molecules, highlights the abundant and tremendous potential of the marine environment as a resource in the field of drug development [11]. Importantly, over the past few decades, secondary metabolites sourced from coral-associated fungi have demonstrated significant efficacy against various clinical targets [12,13]. In order to screen and identify these compounds, several screening methods are being employed. The most common approach for BLI discovery includes production of an artificially designed inhibitor using chemical synthesis that is based on the scientific knowledge amassed thus far [7,14]. Consequently, testing its properties with various platforms and improving its affinity to the desired β-lactamase via chemical modifications are performed. A more traditional approach involves the high-throughput screening (HTS) of natural components to identify potential BLIs [15]. This approach suggests that natural products produced by microorganisms may contain specific active molecules that are a result of natural selection [16]; however, it requires costly specialized equipment. Another traditional method is nuclear magnetic resonance (NMR) technology, which has the limitation of exploring inhibitors from only a specific class of β-lactamase [14]. To date, assays such as phage display [17] select peptides from a random sequence library [18] and screen for only specific candidates. The fragment-based drug discovery (FBDD) approach focuses on small fragments binding weakly to enzymatic targets in the hope of finding a target with a higher affinity [19,20]. These are considered to be time-consuming and costly [21]. A complementary advanced alternative to the FBDD method is the utilization of computer-based virtual screening and a deep learning approach that only focuses on the modification of known compounds [14]. The flaws and drawbacks of the above-mentioned methods reflected in the complex technological and laboratory systems mentioned above are hampering quick identification. Therefore, with the aim to advance a practical, efficient, easy-to-use, and sensitive tool for BLI identification, we developed a relatively simple, innovative, and sensitive method that includes a bioluminescence-dependent bioassay platform. This is because whole-cell biosensors retain high specificity, the ability to detect multiple analytes simultaneously, and the potential for rapid and cost-effective detection [22,23]. It should be kept in mind that this innovative assay may provide a preliminary proof of concept of β-lactamase inhibitors present in various environmental samples.

Furthermore, such a bioassay will allow assessing the potential of naturally occurring active compounds, such as β-lactamase inhibitors, that in the future may be further structurally identified using analytical methods.

## 2. Materials and Methods

### 2.1. Materials

β-lactam antibiotics (BLAs), piperacillin, ampicillin, kanamycin, chloramphenicol, and penicillin G were purchased from Sigma-Aldrich (Rehovot, Israel), and the β-lactamase inhibitors (BLIs), tazobactam and sulbactam, from Abcam (Cambridge, UK). All were diluted in distilled deionized water (DDW) and stored at the recommended temperatures by the manufacturer.

### 2.2. Bacterial Strains

Four different transformed *E. coli* bacteria containing a stress-response plasmid and a resistant plasmid are used for this bioassay (Table 1). The stress plasmid includes the fusion of one of two stress promoters to the *lux* gene originating from *Vibrio fischeri* as a bioluminescent reporter [24], enabling real-time monitoring of the bacterial response through a straightforward luminometry technique. This assay uses luciferase light emission via the *lux* gene to report exposure to genotoxic or cytotoxic stress by bioluminescence. The stress promoter is either the SOS *recA* promoter, which is triggered by genotoxic damage that induces DNA damage repair systems activity [25], or the heat-shock *grpE* promoter that is activated in cytotoxic damage where metabolic changes occur due to temperature alteration, oxidative stress, and exposure to toxic substances [22,26]. The resistant plasmid includes one of two plasmids that either contain the *bla*_CTX-M-2_ gene [27] or the *bla*_SHV-12_ gene [28].

### 2.3. Strain Growth Conditions

Bacterial cultivation was carried out in Luria–Bertani (LB) medium supplemented with a mixture of 50 mg/mL of kanamycin and 100 mg/mL of penicillin G. Cells were grown overnight at 37 °C in a rotary thermo-shaker (Gerhardt, Königswinter, Germany) at 120 rpm. Prior to the experiment, the bacterial medium was diluted one hundred-fold in a fresh medium and was re-grown at 30 °C, without shaking and without an antibiotic addition to reach an early exponential phase (OD_595_ = 0.2).

### 2.4. Bioluminescence Assay

The samples were placed in a white opaque 96-well microtiter plate (DanyelBiotech©, Rehovot, Israel) and the bioluminescence was measured at 490 nm using a Luminoskan Ascent luminometer (ThermoFisher Scientific, Waltham, MA, USA). The bioluminescence values are presented in relative light units (RLUs) and were measured at 5 min intervals for 12 h at 26 °C. Each well contained 80 µL of the bioreporter strain, 10 µL of BLAs, and 10 µL of a BLI candidate. DDW was used as a negative control, and 800 ppb mitomycin C and 2% (*v*/*v*) ethanol were used as positive controls for E001, E002 and E003, E007, respectively. In the preliminary experiment, to ascertain possible background bioluminescence of each component, BLA or BLI candidates were tested separately. To calculate the tested compound’s effect on the bioreporter strains, the maximal RLU signals were normalized by the maximal RLU of the control, and this ratio was referred to as the induction factor (IF). The IF was calculated using the following formula:

$$IF=\frac{Bi}{BC}$$

Bi is the maximum bioluminescent signal obtained from either the commercial BLI or BLA, and BC is the maximum signal obtained from the control.

To measure the percent inhibition of bioluminescence, the maximal bioluminescence response to the BLA-BLI combination was normalized by the maximal RLU signal obtained from BLAs and BLIs separately. Inhibition of bioluminescence by fungal BLIs was calculated according to the following formula:

$$BLI=\left(1-\frac{fungal\;supernatant+BLA}{fungal\;supernatant\times BLA}\right)\times 100$$

Inhibition of bioluminescence by fungal supernatant as an indication for antimicrobial activity (AM) was calculated according to the following formula:

$$AM=\left(1-fungal\;supernatant\right)\times 100$$

Positive inhibition percentages indicate bioluminescence repression due to the addition of the BLI, while negative values indicate that BLIs led to bioluminescence activation. Values in the range of zero imply that the BLI did not affect the bacteria’s bioluminescence.

### 2.5. Optimization of Bacterial Response to β-Lactam Antibiotics

The highest concentration of antibiotics that does not cause changes in luminescence was determined for each bacterial strain. This concentration served as the threshold concentration, indicating that the bacteria are actively producing β-lactamase through their β-lactam resistance plasmid without experiencing any DNA or protein damage. To establish the threshold concentration, a gradient of six dilutions of the antibiotic was prepared. Due to the solubility properties of the antibacterial agents in DDW, the highest concentrations of 50 mg/mL for ampicillin and piperacillin were selected.

### 2.6. Optimization of the Bioassay Using Commercial β-Lactamase Inhibitors

To establish the bioluminescence bioassay, the impact of well-known commercial β-lactamase inhibitors (BLIs), sulbactam and tazobactam, was examined. Initially, the individual impact of each BLI on the bioreporter panel was examined to determine the highest concentrations that do not induce stress responses in the bacteria. BLIs were serially ten-fold diluted in DDW, based on their respective 50% inhibitory concentrations (IC50) [30]. The concentrations tested for each BLI were as follows: sulbactam: 0.002–20 µM (IC50 = 0.02–1.9 µM); tazobactam: 0.01–100 µM (IC50 = 0.001–0.9 µM). Following this preliminary assay, three concentrations of each BLI were selected to assess their effects in combination with the corresponding BLAs on the bioreporters. These experiments served to validate the bioassay and its applicability. The BLA-BLIs of ampicillin–sulbactam and piperacillin–tazobactam combinations were selected based on their extensive use in clinics since they effectively restore the antibiotic activity [31].

### 2.7. Collection of Coral-Associated Fungi

Three fragments from two colonies each of the stony corals *Pocillopora* sp., *Acropora* sp., and *Stylophora pistillata* were collected at a depth of 15 m on the North beach of Eilat (29°32′ N 34°58′ E). A total of 18 samples were obtained, with three 5 cm^3^ fragments collected from each coral colony. The samples were fractionated into mucus, soft tissue, and skeleton fractions. For the mucus fraction, the coral samples were centrifuged at 2675 g for 3 min, rested for 30 min, and centrifuged again under the same conditions to remove all secreted mucus. The soft tissue was separated from the coral using an airbrush with filtered seawater (0.22 µm membrane), and the denuded skeleton was then crushed using a mortar and pestle. Cultivation of fungal samples was conducted by plating samples from the mucus, soft tissue, and skeleton fractions onto Petri dishes with various media: Potato Dextrose agar (PDA) (HIMEDIA), Glucose Peptone Yeast extract (GPY) (glucose of 20 g, yeast extract of 10 g, peptone of 20 g, pH 6.5), Czapek-Dox (NaNO_3_ of 3 g, KCl of 0.5 g, K_2_HPO_4_ of 0.1 g, MgSO_4_·7H_2_O of 0.5 g, FeSO_4_ of 0.01 g, sucrose of 30 g, agar of 20 g, pH 6.7), Malt extract (HIMEDIA), and Sabouraud dextrose (Difco) [32]. All media were prepared with 1 L of artificial seawater (ASW) (NaCl of 24.6 g, KCl of 0.67 g, CaSO_4_·7H_2_O of 6.29 g, MgCl_2_·6H_2_O of 4.66 g, NaHCO_3_ of 0.18 g, DDW of 1 L, pH 7.5), with the addition of chloramphenicol antibiotic of 250 mg/L.

The plates were incubated at 26 °C for 1–3 weeks until fungal colonies appeared. Thirteen coral-associated fungi were isolated.

### 2.8. Fungal DNA Extraction and Sequence Analysis

Genomic DNA of the isolated coral-associated fungi was extracted using the PowerSoil purification kit (MoBio). The fungi were identified based on the ITS sequence (ITS1-5.8S-ITS2) using ITS1-F (5′-CTTGGTCATTTAGAGGAAGTAA-3′) [33] and ITS4 (5′-TCCTCCGCTTATTGATATGC-3′) [34] primers. The PCR reaction program was as follows: pre-heating at 95 °C for 4 min, 28 cycles of denaturation at 94 °C for 25 s, annealing at 55.5 °C for 30 s, extension at 72 °C for 1 min, and final extension at 72 °C for 2 min. Sequencing was performed using the ABI PRISM dye terminator cycle sequencing ready reaction kit with AmpliTaq DNA polymerase FS and an ABI model 373A DNA sequencer (Perkin-Elmer).

In total, 18S rRNA gene sequences were compared with those in the GenBank database using the basic alignment search tool BLAST, and a representative taxon was chosen for each fungus (Table 2).

### 2.9. Coral-Associated Fungal Processing

The isolated fungi were cultivated on agar plates for a period of 1–2 weeks prior to growing in a liquid medium with agitation at 30 °C for 7 days. In total, 2 mL of the fungal cultures was filtered using membranes with a pore size of 0.22 µm and subsequently stored at 4 °C. These were subsequently tested using the bioreporters.

### 2.10. Using the Developed Screening Platform to Test Coral-Associated Fungi as BLI Producers

Based on the reproducibility and highest bioluminescence signals, two bioreporter strains, E002 and E003, were selected for further screening for natural BLIs. Fungal cell-free cultures were assessed as a potential source for the β-lactamase inhibitor (BLI), and commercial BLIs were used as positive controls. Prior to the screening procedure, the response of the bioreporter strains to the sterile fungal media was examined to exclude any sensitivity related to the salts present in the media. Based on the results of these preliminary experiments, all media containing fungal agents and the sterile media used as controls were diluted to a 1:100 ratio in DDW.

## 3. Results

### 3.1. Bioassay Optimization, Validation, and Proof of Concept

The first stage of the bioassay development was assessing the bioreporter responses to each of the commercially used BLAs or BLIs individually. The threshold concentrations for each antibiotic were determined as follows: for *E. coli* strains E001 and E007, the ampicillin threshold concentration was set at 50 µg/mL, while for *E. coli* strains E002 and E003, it was 5 µg/mL (Figure S1, Table S1). Regarding piperacillin, the threshold concentration was 5 µg/mL for *E. coli* strains E001 and E003, 50 µg/mL for the E007 strain, and 0.5 µg/mL for the E002 bioreporter (Figure S2, Table S1). The second optimization step included the outcome response of the corresponding inhibitor (BLI) on the bioreporters. This was carried out in order to identify the concentrations that either had no significant effect or had minimal impact on the bioreporters themselves. Overall, the bioreporter *E. coli* E001 and E007 strains, which contained the CTX-M-2 resistance gene, remained unaffected by the BLIs, even at the highest tested concentrations. However, the bioreporter *E. coli* E002 and E003 strains, which contained the SHV-12 resistance gene, exhibited reduced resistance at one time point during the experiment when the luminescence levels increased (Figures S3 and S4).

CTX-M and SHV beta-lactamases differ significantly in their substrate hydrolysis profiles and inhibitor susceptibility. SHV enzymes show varying degrees of susceptibility to clavulanate, tazobactam, and sulbactam, where CTX-M enzymes are generally more susceptible to tazobactam than clavulanic acid [35,36].

**Figure 1:**
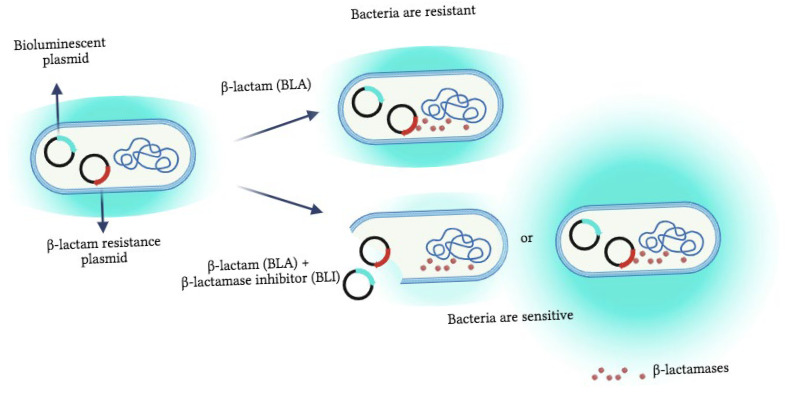
Schematic representation of the response of the bioluminescent reporter strain to the presence of the β-lactamase inhibitor. Unless the BLI is present, the bacteria are resistant in the milieu with β-lactam antibiotics (BLAs) because of the resistance plasmids. However, when the BLI is added, the sensitivity towards antibiotics is restored, and the bioreporter may start to die, as denoted by a decreasing bioluminescence signal. It is also possible that the concentration of the antibiotic itself is sub-lethal; therefore, the stress-response promoters can be activated, and the bioluminescence reaction increases. Created using BioRender.com.

**Figure 2:**
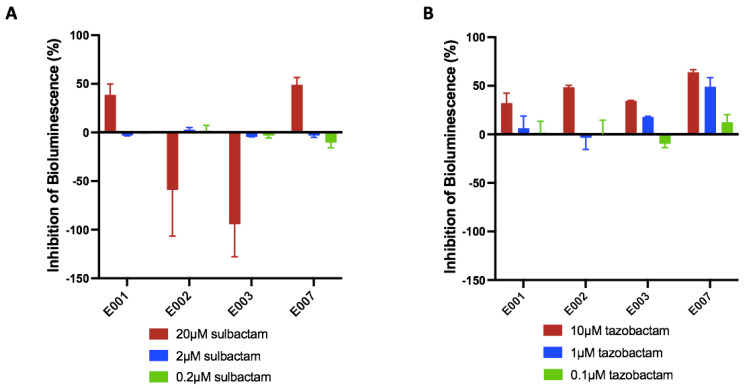
Inhibition percentage of bioluminescence produced by bioreporter strains (E001, E002, E003, and E007) in response to commercial antibiotics and three concentrations of corresponding BLIs. Two BLA-BLI combinations are presented: (**A**) ampicillin-sulbactam; (**B**) piperacillin-tazobactam. The BLA and BLI concentrations: ampicillin: 50 µg/mL (E001, E007) and 5 µg/mL (E002, E003); sulbactam: 20 µM, 2 µM, and 0.2 µM; piperacillin: 0.5 µg/mL (E001, E002) and 5 µg/mL (E003, E007); tazobactam: 10 µM, 1 µM, and 0.1 µM. (*n* = 3 for each fungal supernatant).

Finally, the proof-of-concept step included an optimization stage evaluating the influence of BLA-BLI combinations on the bioreporter’s luminescence, reflecting their stress response. Two optional responses towards the BLA-BLI combination could be recorded: an unaffected bioluminescence signal or altered bioluminescence characterized by either activation or repression (Figure 1 and Figure S5 ). The example shown in Figure S5 demonstrates that bacteria are resistant to the presence of ampicillin or sulbactam alone (minor changes in the IF over time); however, a combination of both stimulates the bioluminescence of the E002 strain. An opposite trend was obtained with E007, where bioluminescence repression was determined with the tested BLA-BLIs (Figure S5). The bioassay sensitivity was assessed through experiments involving various combinations of BLA-BLIs with the E. coli bioreporters panel (Figure 2). It provided insights into the impacts of three concentrations of BLIs in concurrence with the corresponding BLAs on the bioreporter strains. The resulting bioluminescence signals were presented as inhibition percentage, where positive inhibition values designated bioluminescence repression while negative values indicated bioluminescence activation due to the BLI addition. The response of the bacteria to the presence of the ampicillin–sulbactam combination was categorized into two groups based on the specific resistance gene carried by the bacteria (Figure 2A).

Bioreporter *E. coli* strains E001 and E007, which both contain the gene *bla_CTX-M-2_*, exhibited decreased bioluminescence of approximately 50% at the highest sulbactam concentration of 20 µM. On the other hand, E002 and E003 strains, carrying the gene *bla_SHV-12_*, showed increased bioluminescence, negative values of 60% and 90%, respectively, for the highest concentration of sulbactam. In the piperacillin–tazobactam combination, all four *E. coli* bioreporter strains demonstrated similar positive bioluminescence responses (Figure 2B). Notably, higher concentrations of tazobactam resulted in a stronger bioluminescence repression. A negative percent of bioluminescence means that the antibiotic does not kill the bioreporter strains; thus, the rationale is to find combinations of BLA-BLIs that, on the one hand, do not kill the bioreporters and, on the other hand, can activate their promoters. Overall, the bioluminescence enhancement or depression varied as a function of BLI concentration.

### 3.2. Detection of Potential β-Lactamase Inhibitor Agents from Fungal Isolates

**Figure 3:**
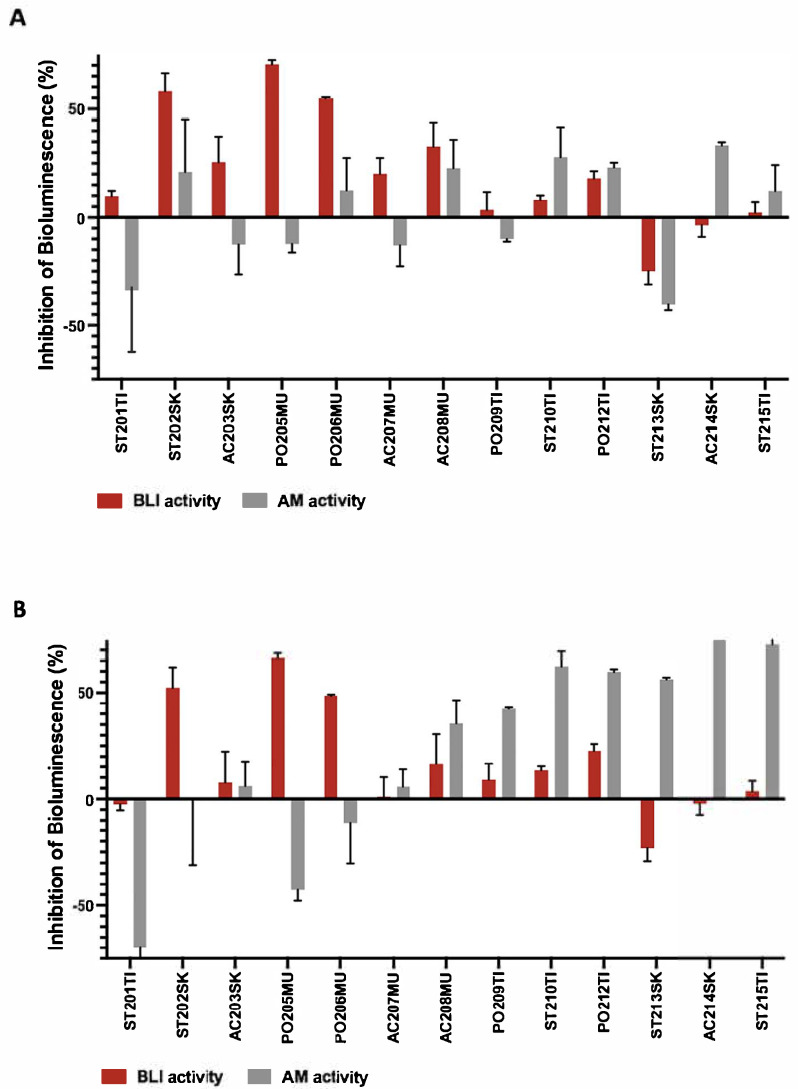
Detection of ponteal BLIs and antimicrobial activity of components derived from the coral-associated fungi supernatant using the bioluminescence bioassay. Antibiotic activity is calculated by the fungal supernatant effect alone. (**A**) Bioreporter E003, 5 µg/mL of ampicillin. (**B**) Bioreporter E002, 0.5 µg/mL of piperacillin (*n* =3 for each fungal supernatant). AM activity designated the potential antimicrobial activity by the fungal supernatant.

Cell-free cultures derived from the 13 coral-associated fungi were screened for their inhibitory function of β-lactamase using the novel bioassay. The BLI activity of the cell-free fungal culture was tested using the *E. coli* bioreporter strain E003 supplemented with ampicillin (Figure 3A) and E002 supplemented with piperacillin (Figure 3B). Fluctuations in the bioreporter responses to fungal supernatants combined with the corresponding antibiotic were normalized to the antibiotic effect alone and the fungal supernatant effect alone. Bioluminescence inhibition percentage values above or below 10% indicated the potential presence of BLIs secreted by the fungi. The fungal isolate screening revealed that the addition of the ST202SK, PO205MU, and PO206MU supernatants decreased the E003 bioreporter strain response against ampicillin (Figure 3A). A similar response was observed with the *E. coli* E002 strain towards the piperacillin when ST202SK, PO205MU, and PO206MU were added (Figure 3B). These three fungal isolates presented the most significant bioluminescence inhibition above 50%.

To eliminate the antibacterial properties of the fungal cell-free cultures, the bioreporter response to the fungal supernatant alone was also examined (Figure 3A,B). The results demonstrated antimicrobial activity for three fungal strains (ST201TI, AC214SK, and ST215TI) that retained the potential for secreting antibacterial components. However, these isolates did not overlap with the BLI-secreting isolates. Therefore, isolates were considered potential BLI production candidates when significant bioluminescence changes were observed between supernatants with antibiotics added to the supernatants alone, above 10% of change.

## 4. Discussion

The marine habitat is perceived as a gold mine of active metabolites. Approximately half of the clinically approved drugs are originally derived from natural products produced by living organisms, in particular those of marine origin [37,38]. Marine fungi have recently become recognized as possible sources of novel bioactive materials [12,39]. Indeed, a great structural diversity was found in the 423 metabolites that were isolated from coral-associated fungi between 2010 and 2021 [13. Furthermore, some of them were found to possess a wide range of bioactivities, including anticancer, antimicrobial, and antifouling activities [13]. These molecules are being harnessed to fight antibiotic resistance that is becoming more and more prevalent today. The extensive use of antibiotics, including β-lactams (i.e., penicillin, cephalosporins, monobactams, and carbapenems), which are the most prescribed group, has led to resistance primarily via β-lactamase-mediated dissemination [2,3,40]. It was found that novel BLIs (β-lactamase inhibitors), when combined with β-lactam antibiotics (BLAs), can effectively combat antibiotic resistance through the protection of the antibiotic structure from degradation by beta-lactamase enzymes [3]. Interestingly, BLI activity has been found in natural products derived from a variety of plants [41,42] and fungi [43,44]. Indeed, in recent years, a few non-conventional BLA-BLIs, comprising a unique class of BLIs that do not contain the characteristic core structure of a beta-lactam ring in combination with extended-spectrum cephalosporin or carbapenem, have entered the market (e.g., avibactam, vaborbactam, and relebactam). One such combination, Ceftazidime–avibactam, was approved by the FDA in 2015 for treating complicated urinary tract (cUTI), complicated intra-abdominal infections (cIAIs), and hospital-acquired pneumonia (HAP), including ventilator-associated pneumonia (VAP) [45]. Additionally, Meropenem–vaborbactam was approved in 2017 for cUTI and pyelonephritis, and imipenem–cilastatin–relebactam was certified in 2020 for the treatment of cUTI, cIAI, and HAP/VAP [46,47,48].

In light of the importance of BLIs in maintaining antibiotic effectiveness, we searched for additional sources of BLIs. The present study’s design resulted from previous findings that showed the presence of the β-lactamase gene in biofilm-associated marine bacteria [49] as well as in planktonic marine microbial assemblages [50]. We developed a bioassay that could detect the presence of possible BLIs in compounds eluted from fungal isolates from marine environments. The newly developed bioassay in this study is based on a panel of two out of four genetically engineered *E. coli* strains, each possessing two plasmids. These plasmids provide the bacteria, on the one hand, with the ability of β-lactamase synthesis in the presence of β-lactam antibiotics and, on the other hand, a plasmid-mediating bioluminescence response towards stress markers. In the developed bioluminescence-based assay, two components of a commercial antibiotic together with a BLI are introduced to the bioreporters. The antibiotic is added to the bioreporter at a concentration below its threshold to avoid triggering a bacterial stress response. Therefore, any change in the bioluminescence pattern reflects the effect of the tested mixture. During the screening process, the bioluminescent signal emitted from the bioreporter may increase, decrease, or remain unaffected. In the case of no change, the tested mixture probably does not function as a β-lactamase inhibitor (BLI) or its concentration is insufficient. A significant increase or decrease in the bioluminescence signal indicates BLI activity. The innovative bioassay validated here, by using predetermined concentrations of BLAs and BLIs, where BLAs alone did not activate the bioreporter bacteria, and only following the addition of commercial BLIs, showed that the system is stimulated in a detectable and analyzable manner. Moreover, the bioassay proved to be sensitive to different concentrations of the BLI and yielded outcomes depending on the combination of the BLI type and the bioreporter bacteria used (Figure 2). Following the bioassay validation using commercial BLIs, coral-associated fungal isolates were screened for possible β-lactamase inhibition agents (Figure 3). The assay proved to be amenable to the detection of the fungal-derived compounds. Therefore, this assay provides additional evidence that the dense microbial population on or in the model coral is likely to produce β-lactamase inhibitors. Although β-lactamase production is primarily known to be found in bacteria, in this study, we showed that fungi may also produce BLIs as a defense mechanism and as a strategy to compete for resources. We compared the bioreporter bacteria’s response to fungal supernatant alone, to ampicillin alone, and to the mixture of both. A minor effect was noticed with ampicillin or piperacillin alone in the selected concentrations for fungal supernatant assessment, since the bioreporter has a resistance gene for β-lactamase synthesis. The addition of fungal supernatant to the antibiotic caused several different responses (Figure 3). First, when the change in bioluminescence was above 10%, the fungal supernatant was considered potentially active as a BLI. The ST213SK isolate showed a negative percentage change lower than 10%. Samples with positive bioluminescence inhibition were almost all higher than 10%, excluding ST201TI, ST213SK, AC214SK, and ST215TI as indicated by the E002 and E003 strains.

To confirm that the fungal supernatant does not contain antibacterial products, the bacterial response to the fungal supernatant alone was also tested (Figure 3, AM activity).

When similar values of bioluminescence inhibition were obtained for both, fungal supernatant alone, and that with the addition of BLAs, as can be seen for ST201TI, AC214SK, and ST215TI isolates, this demonstrated that the fungus may have produced an antibiotic and not BLIs.

Non-similar values of bioluminescence inhibition indicate that the fungal supernatant poses potential BLI activity, as was determined for ST202SK, PO205MU, and PO206MU isolates by E002 and E003 bioreporters (Figure 3A,B). Taken together, the results suggest a significant advantage of the proposed bioassay as reflected by the ability to differentiate between antibacterial activity and BLI activity in the same experiment, making the screening platform specific and capable of simultaneously monitoring BLIs and new antibacterial components, thus effectively distinguishing between signals.

Several platforms for BLI assays with varying throughput, sensitivity, and application capabilities have been reported over the years. For example, cephalosporin-based substrates that undergo changes in their UV or visible light absorbance, as their structure is altered by the cleavage of their β-lactam ring by β-lactamase are in use, but these substrates, including nitrocefin [51], are expensive and require multi-step synthesis processes. Other chromogenic substrates like PADAC [51], CENTA [52], chromacef [53], and CLS405 [54] may suffer from lower sensitivity to enzyme hydrolysis, low stability, and light sensitivity. There is also a platform that utilizes a fluorogenic cephalosporin-based substrate that is hydrolyzed by β-lactamase and is monitored through fluorescence signal, offers superior sensitivity and kinetic parameters compared to chromogenic ones [55,56]. They share a disadvantage of being suitable only for screening for BLIs that are optimally matched with the specific cephalosporin-based substrate but not with other BL antibiotic families.

Unlike the platforms available today, using the bioluminescence bioassay proposed here offers the advantage of being able to screen both synthetic molecules and natural products for BLI discovery. Furthermore, our bioassay directly tests BLI candidates using bacteria, enabling the discovery and focus on compounds that ultimately can evolve into pharmaceutical inhibitors. The bioreporter system described here was shown to be amenable for identifying BLI activity in a concentration-dependent manner. Furthermore, unlike other screening assays, the bioreporter panel of the newly developed bioluminescence assay can be expanded with various isolated beta β-lactamase genes, which will determine the type of added antibiotic and the potential BLI. Furthermore, a notable benefit of this bacterial luciferase-based bioassay is its ability to express an entire luciferase operon, generating a luminescent cell without any external substrate addition. This feature enables the real-time monitoring of gene expression.

In summary, a bioluminescence-dependent bioassay was developed for detecting new BLI candidates. Since the bioassay is based on *E. coli* bioreporters and requires only small volumes of reagents, the bioassay is cost-effective. In addition, the bioassay was found to be sensitive even to low concentrations of β-lactamase inhibitors as presented with common combinations of commercial drugs (≥0.2 µM of sulbactam; ≥0.1 µM of tazobactam). The clinical applications of these inhibitors currently act mostly against class A β-lactamase. Therefore, the bioreporters chosen in our panel include two common resistant plasmids against class A β-lactamase (*bla*_CTX-M-2_ and *bla*_SHV-12_). This bioreporter panel can potentially be expanded with additional bioreporters, engineered with plasmids that contain genes encoding β-lactamases from other classes, such as *bla*_IMP_ (class B) or *bla*_oxa_ (class D), for more extensive screening and be used effectively in high-throughput screening. This present study offers an innovative bioluminescence-based assay that shows potential BLI activity from coral-associated fungi. This is not a stand-alone assay, and structural analysis of the screen molecules will be required for further BLI validation.

## Figures and Tables

**Table 1:** Bioreporter *E. coli* strains used in this study [29].

Strains	Bioluminescent Plasmids	β-Lactam Resistance Gene
**E001**	recA::*lux*	*bla*_CTX-M-2_
**E002**	recA::*lux*	*bla*_SHV-12_
**E003**	grpE::*lux*	*bla*_SHV-12_
**E007**	grpE::*lux*	*bla*_CTX-M-2_

**Table 2:** Fungal isolate identification from the coral using the ITS1-5.8S-ITS2 region. The isolate name indicates its source: the first two letters of the isolate name represent the host coral’s fungal origin (ST = Stylophora, PO = Pocillopora, AC = Acropora). The last two letters indicate the coral layer source of the fungus (MU = mucus, SK = skeleton, TI = soft tissue). The closest relatives to fungal strains according to the NCBI BLAST are presented.

Isolate Name	Source Coral Genus	Medium	Most Related Strains by BLAST + Accession Number + Identity (%)
**ST201TI**	*Stylophora* (coral No. 1)	GPY + ASW	*Cladosporium sphaerospermum * KP701988.1 (100%)
**ST210TI**	*Stylophora* No. 1	PDA + ASW	*Cladosporium sphaerospermum * KP701988.1 (100%)
**PO209TI**	*Pocillopora * No. 1	PDA + Bupirimate + ASW	*Cladosporium sphaerospermum * KP701988.1 (100%)
**ST202SK**	*Stylophora * No. 2	GPY + ASW	*Aspergillus terreus * KC119206.1 (100%)
**ST213SK**	*Stylophora* No. 2	Czapex-Dox + ASW	*Aspergillus terreus * KC119206.1 (100%)
**AC203SK**	*Acropora* No. 1	Malt Extract + ASW	*Cladosporium cladosporioides * AJ300335.1 (100%)
**PO205TI**	*Pocillopora* No. 1	GPY + ASW	*Alternaria alternata * KU182490.1 (100%)
**PO206MU**	*Pocillopora* No. 2	GPY + ASW	*Penicillium spinulosum * KF646101.1 (99%)
**ST215TI**	*Stylophora* No. 2	Czapex-Dox + ASW	*Penicillium spinulosum * KF646101.1 (100%)
**AC207MU**	*Acropora* No. 2	Malt Extract + ASW	*Cladosporium halotolerans * KP701958.1 (100%)
**AC208MU**	*Acropora* No. 2	Malt Extract + ASW	*Cladosporium perangustum * KP701968.1 (99%)
**PO212TI**	*Pocillopora * No. 1	PDA+ ASW	*Cladosporium cladosporioides * KU182497.1 (100%)
**AC214SK**	*Acropora* No. 1	Czapex-Dox + ASW	*Cladosporium cladosporioides * KU182497.1 (100%)

## Data Availability

All data generated or analyzed during this study are included in this published article and its Supplementary Information file. The strains, reagents, and datasets used and/or analyzed during the current study are available from the corresponding author on reasonable request.

## Supplementary Materials

The following supporting information can be downloaded at https://www.mdpi.com/article/10.3390/bios15090594/s1, Figure S1: Bioluminescence response to gradient of ampicillin concentrations solely; Figure S2: Bioluminescence response to gradient of piperacillin concentrations solely; Figure S3: Bioluminescence response to gradient of sulbactam concentrations solely; Figure S4: Bioluminescence response to gradient of tazobactam concentrations solely; Figure S5: Activation versus repression of damage gene; Table S1: Summary of the chosen antibiotic concentrations, which were considered as the threshold concentrations.

## Abbreviations

The following abbreviations are used in this manuscript:

XDR	Extensive drug-resistant
MDR	Multidrug-resistant
BLAs	β-lactam antibiotics
PBPs	Penicillin-binding proteins
ESBLs	Extended-spectrum β-lactamases
BLI	β-lactamases inhibitor
BLA	β-lactam antibiotic
MICs	Minimum inhibitory concentrations
LB	Luria–Bertani
RLU	Relative light units
DDW	Double-distilled water
IC50	50% inhibitory concentrations
PDA	Potato Dextrose agar
GPY	Glucose Peptone Yeast
ASW	Artificial seawater
cUTI	Complicated urinary tract
cIAI	Complicated intra-abdominal infections
HAP	Hospital-acquired pneumonia
VAP	Ventilator-associated pneumonia
HTS	High-throughput screening
NMR	Nuclear magnetic resonance
FBDD	Fragment-based drug discovery
