# Supplementary material for: Whole-Cell Bioreporter-Based Assay for Detecting Fungal-Derived β-Lactamase Inhibitors
Source: Biosensors (Basel). 2025 Sep 9;15(9):594. doi: 10.3390/bios15090594 (PMC12467308; doi:10.3390/bios15090594)
Supplement: Supplementary file 1 [file biosensors-15-00594-s001.zip › biosensors-3705272-supplementary.pdf]

Supplementary

Optimization of Bioreporter Response to β-Lactam Antibiotics

A gradient of six to eight antibiotic dilutions were prepared by ten-fold serial dilutions with DDW.

In the optimizations steps, bioreporter response to the BL antibiotics presents as the IF, calculated by dividing the average RLU of wells containing bioreporter with antibiotic by the average RLU of control wells containing the bioreporter without antibiotic but with DDW to complete the volume.

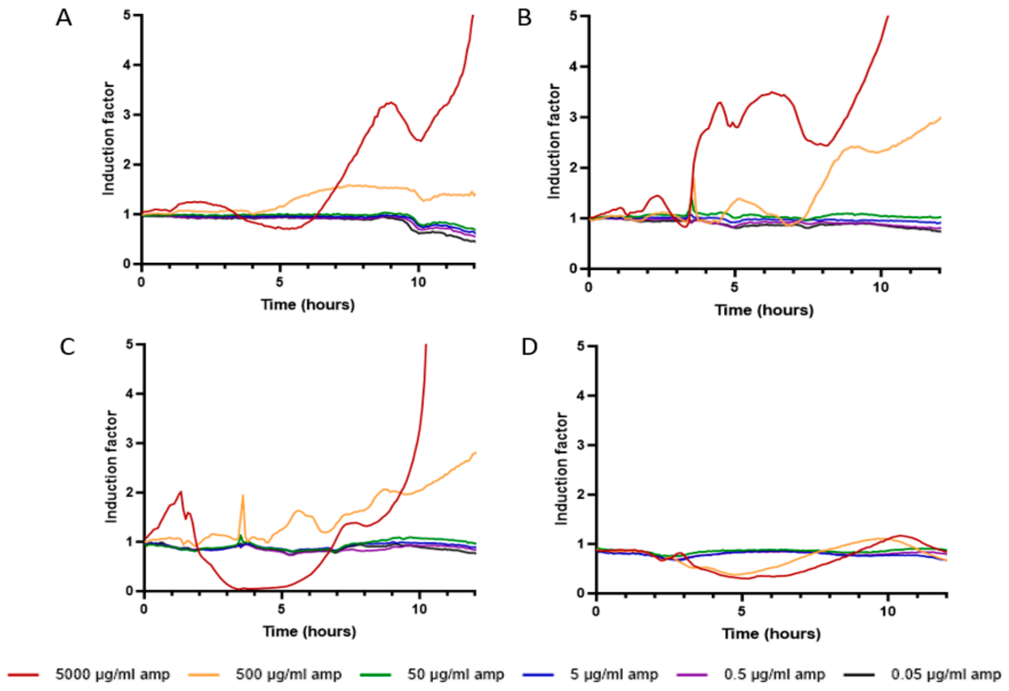

Figure S1. . Bioluminescence response to gradient of ampicillin concentrations solely. (A) E001 bacteria; (B) E002 bacteria; (C) E003 bacteria; (D) E007 bacteria. The selected concentration is the one that did not not arise bioreporter luminecence: (A) 50 µg/ml; (B) 5 µg/ml; (C) 5 µg/ml; (D) 50 µg/ml.

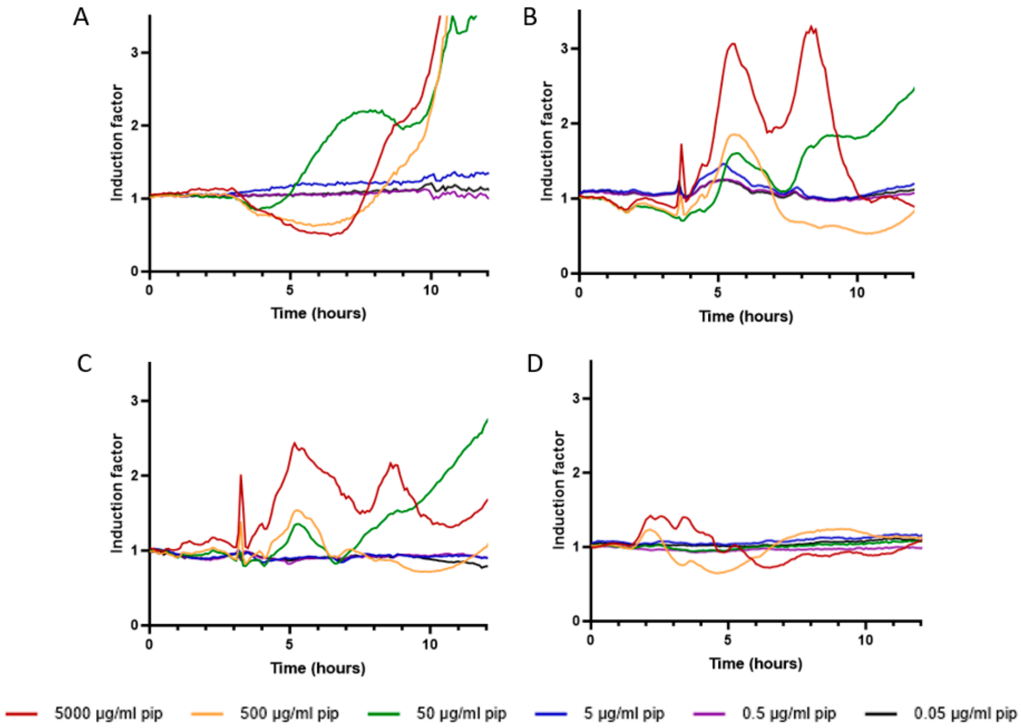

Figure S2. Bioluminescence response to gradient of piperacillin concentrations solely. (A) E001 bacteria; (B) E002 bacteria; (C) E003 bacteria; (D) E007 bacteria. The selected consentration is the one that did not not arise bioreporter luminecence: (A) 5 µg/ml; (B) 0.5 µg/ml; (C) 5 µg/ml; (D) 50 µg/ml.

Optimization of Bioreporter Response to Commercial β-Lactamase Inhibitors

A gradient of five BLIs' dilutions were prepared by ten-fold serial dilutions with DDW. In optimizations of bioreporter response to the BLIs, IF was calculated by dividing the average RLU of wells containing bacteria with BLIs by the average RLU of control wells containing the bacteria without antibiotic but with DDW to complete the volume.

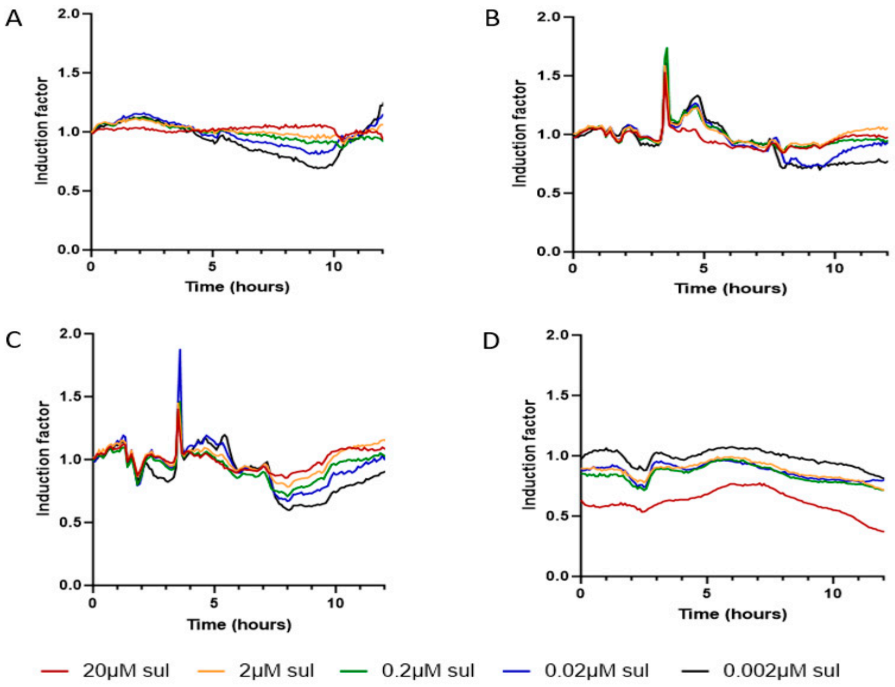

Figure S3. Bioluminescence response to gradient of sulbactam concentrations solely. (A) E001 bacteria; (B) E002 bacteria; (C) E003 bacteria; (D) E007 bacteria.

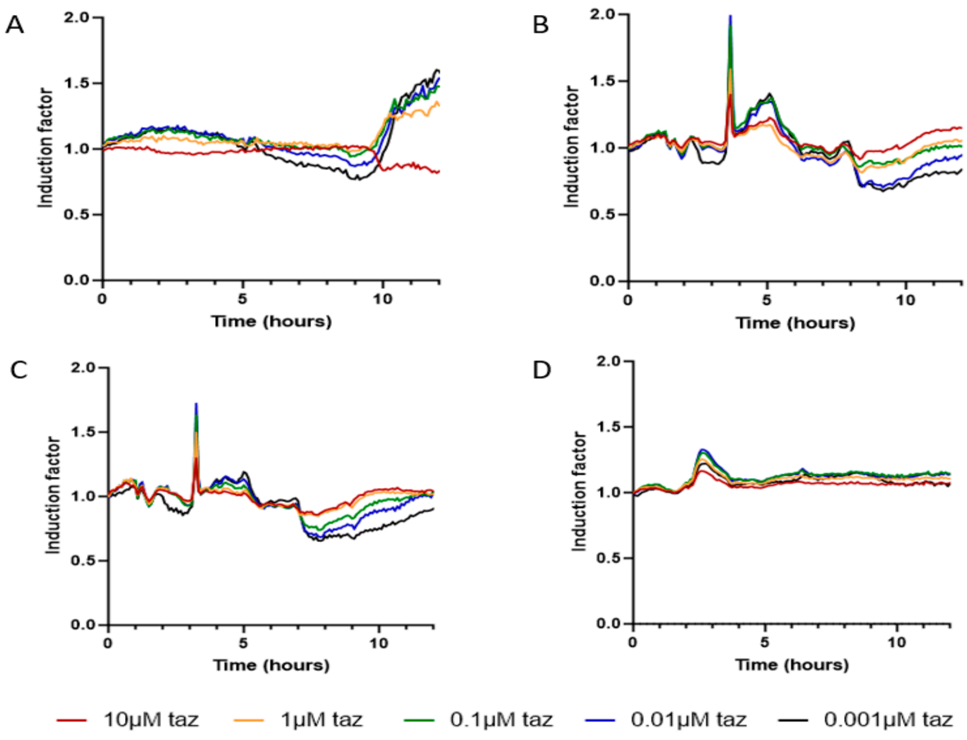

Figure S4. Bioluminescence response to gradient of tazobactam concentrations solely. (A) E001 bacteria; (B) E002 bacteria; (C) E003 bacteria; (D) E007 bacteria.

Activation and Repression of Bioluminescence

Bacterial strains E002 and E007 are resistant to the presence of ampicillin or sulbactam singly, demonstrated by minimal changes in the IF value through time and that the combination of ampicillin with sulbactam stimulated luminescence. Examples of bioluminescence activation and repression can be seen in figure 5SA and 5SB, respectively .

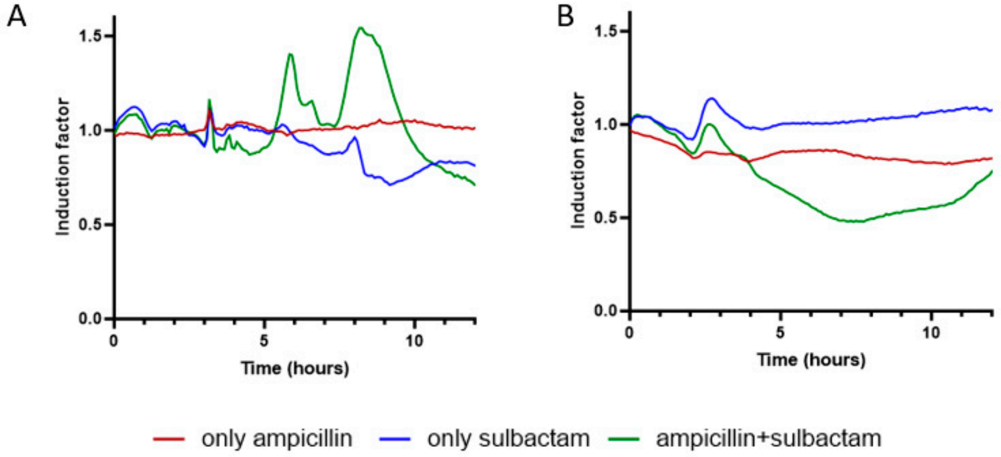

Figure S5. Activation versus repression of damage gene. Bioreporter bacteria response to presence of ampicillin (BL) alone, sulbactam (BLI) alone and the mixture of both. (A) Bioreporter bacteria E002, 5 µg/ml ampicillin, 20 µM sulbactam (B) Bioreporter bacteria E007, 50 µg/ml ampicillin, 20 µM sulbactam.

Table S1. Summary of the chosen antibiotic concentrations, which considered as the threshold concentrations.

Antibiotic / Bacterial *E. coli* strain	Ampicillin (µg/ml)	Piperacillin (µg/ml)
E001	50	5
E002	5	0.5
E003	5	5
E007	50	50
